# Beyond the Mission: Long-Term Endocrine Dynamics in Search and Rescue Dog–Handler Teams

**DOI:** 10.3390/ani16121934

**Published:** 2026-06-22

**Authors:** Justyna Wojtaś, Klaudia Kaliszyk, Kamila Kaszycka, Piotr Czyżowski, Aneta Strachecka, Patrycja Staniszewska, Bengü Bi̇lgi̇ç, Mehmet Erman Or

**Affiliations:** 1 Department of Animal Ethology and Wildlife Management, University of Life Sciences in Lublin, Akademicka 13, 20-950 Lublin, Poland; justyna.wojtas@up.edu.pl (J.W.); kamila.kaszycka@up.edu.pl (K.K.); piotr.czyzowski@up.edu.pl (P.C.); 2 Department of Invertebrate Ecophysiology and Experimental Biology, University of Life Sciences in Lublin, Akademicka 13, 20-950 Lublin, Poland; aneta.stracheka@up.edu.pl (A.S.); patrycja.staniszewska@up.edu.pl (P.S.); 3 Department of Internal Medicine, Faculty of Veterinary Medicine, Istanbul University-Cerrahpasa, Avcilar, Istanbul 34320, Turkey; bengu.bilgic@iuc.edu.tr (B.B.); ermanor@iuc.edu.tr (M.E.O.)

**Keywords:** search and rescue dogs, hair, cortisol, testosterone, behavior

## Abstract

Search and rescue (SAR) dog–handler teams work under challenging conditions that may influence long-term physiological stress and arousal. Hair steroid analysis provides a reliable measure of chronic endocrine activity in SAR teams. Hair cortisol (HCL) and hair testosterone (HTL) offer non-invasive markers of chronic hypothalamic–pituitary–adrenal (HPA) and (the hypothalamic–pituitary–gonadal) HPG axis activity. This study examined long-term endocrine patterns in SAR dogs and their handlers and explored correlations within and between species. Hair samples were collected from 60 SAR dogs and their handlers. Dog hair was taken from the interscapular region, and human hair from the occipital area. Cortisol and testosterone were extracted using established methanol-based protocols and quantified via ELISA. Dogs showed a mean HCL of 10.974 pg/mg and a mean HTL of 3.008 pg/mg. Female dogs had significantly higher cortisol levels than males, and cortisol tended to increase with age. Testosterone did not differ by sex, breed, or castration status. Handlers showed a mean HCL of 10.874 pg/mg and a mean HTL of 2.925 pg/mg, with no sex differences. However, handler cortisol levels varied significantly by dog breed. Additionally, HCL levels of dogs and their handlers were negatively correlated.

## Simple Summary

Search and rescue dog–handler teams face intense operational demands that can affect their long-term stress and well-being. This study measured two key hormones: cortisol (linked to stress) and testosterone (associated with long-term physiological processes), stored in the hair of 60 rescue dogs and their handlers to assess long-term physiological patterns. We found that female dogs showed higher cortisol levels than males, and handlers’ cortisol levels varied depending on the breed of dog they worked with. Higher long-term cortisol levels in handlers were associated with lower levels in their dogs, suggesting a long-term physiological association within the team. The results suggest that the well-being of search and rescue teams is shaped by both individual traits and the human–dog working relationship. Practically, this suggests that monitoring long-term stress in both dogs and handlers could help improve selection, training, and workload management in search and rescue operations.

## 1. Introduction

Search and rescue (SAR) dogs play a key role in crisis management; therefore, their emotional and physical well-being is a vital factor in the effectiveness of emergency measures. Working under those conditions is demanding for both the dog and the handler, which can lead to increased stress in both the short and long term. Physiological measurements, such as cortisol and testosterone levels, might help assess the stress reaction and arousal levels.

Cortisol is a key indicator of the hypothalamic–pituitary–adrenal (HPA) axis’s activity, which plays a key role in the physiological stress response. The HPA axis regulates a coordinated neuroendocrine response that allows the body to maintain homeostasis under changing environmental conditions. Acute activation of this system leads to a transient increase in cortisol secretion. Cortisol mobilizes energy reserves by increasing blood glucose levels and providing the body with a readily available source of energy. It also improves cardiovascular function by preparing the body for the fight-or-flight response. Furthermore, cortisol suppresses the immune response, conserving energy for immediate survival needs [1]. Although a short spurt in cortisol levels mobilises the body to respond to an immediate threat, a prolonged stress response may lead to metabolic deregulation [2]. Prolonged or repeated activation of the HPA axis can result in dysregulation of glucocorticoid signaling. Chronic exposure to elevated cortisol levels is associated with immunosuppressive effects, as well as changes in metabolic regulation, cognitive performance, and behavioral patterns. These effects are widely described in both medical and veterinary literature and may vary depending on context, species, and individual differences [3,4].

Testosterone secretion is modulated by the hypothalamic–pituitary–gonadal (HPG) axis. Beyond its reproductive functions, testosterone is involved in the regulation of social behaviour, motivation, and physiological adaptation to environmental challenges [5]. Although testosterone has historically been associated with dominance and aggression, studies in dogs suggest that behavioural effects are complex and influenced by multiple factors, including breed and reproductive status [6,7]. The physiological regulation of androgen concentrations may also differ according to sex and reproductive status, which should be considered when interpreting testosterone measurements in working dogs [7,8]. The dual-hormone hypothesis proposes that the relationship between testosterone and behaviour is actively moderated by cortisol levels, where specific hormone ratios may influence stress perception and resilience [9,10]. These interactions suggest that circulating and long-term androgen measures reflect integrated endocrine activity rather than a single-axis HPG output, and should therefore be interpreted within the context of both acute stress reactivity and long-term physiological adaptation in working dogs.

Hormone analysis is a valuable tool for understanding the interactions between physiology, stress, and behaviour in humans and animals. While acute behavioral and circulating hormonal measures reflect short-term stress responses, they are highly sensitive to momentary fluctuations and sampling conditions. In contrast, long-term biological matrices such as hair allow retrospective assessment of integrated endocrine activity over extended periods. The primary methods for measuring hormone levels have historically been saliva and blood samples. Hormones levels in biomatrices such as hair, feathers, fins, scales, nails, and teeth reflect HPA activity over longer periods (weeks or even months), making non-liquid biomatrices useful for estimating chronic stress in animals and humans [11,12]. Studies suggest that hair serves as a retrospective marker of free steroid hormone levels, with concentrations reflecting systemic levels measured over several months [13].

Hair hormone analysis is increasingly used in psychoneuroendocrine research [14]. Hair hormone analysis has many potential applications, including monitoring chronic stress in humans and other animals, predicting disease risk or tracking endocrine disorders (e.g., Cushing’s disease), monitoring psychiatric disorders (e.g., depression and anxiety), and genetic selection or management of animals [12,15]. It is beneficial when we are interested in chronic HPG/HPA patterns without the acute stress of sampling [16]. However, hormone levels in hair can be influenced by factors such as hair growth rate, pigmentation, cosmetic treatments, and environmental pollutants, which should be taken into account when interpreting the results [17].

By now, most work on cortisol–testosterone correlations in dogs has been conducted on pet or shelter dogs [18,19], and the correlations in working dogs, especially in SAR dogs and their handlers, have not been evaluated. Additionally, there is a notable lack of research on testosterone in SAR dogs, as most papers focus solely on cortisol responses.

This study aimed to assess long-term cortisol and testosterone responses in SAR teams by measuring their levels in dogs’ fur and human hair. It was assumed that those levels might be correlated, but they may differ between pairs due to an individual’s past experiences.

## 2. Materials and Methods

The study was approved by the Animal Welfare Committee of the Faculty of Animal Sciences and Bioeconomy of the University of Life Sciences in Lublin, Poland (ZdsDZ/8/2023 of 19 October 2023) and the University Ethical Committee for Research Involving Human Subjects of the University of Life Sciences in Lublin, Poland (UKE/07/2023 of 19 October 2023). Furthermore, all dog handlers were of legal age and provided written consent to participate in the study voluntarily.

The study included 60 rescue teams, consisting of a handler and their dog. Women predominated among the handlers, accounting for 61.7% of participants (*n* = 37), while men accounted for 38.3% (*n* = 23). All handlers owned their dogs, kept them at home, and regularly trained them in specialised skills to prepare them for rescue operations. Additionally, of the 60 handlers, 43 reported having more than one dog at home, while 39 reported that their dog used a kennel. The dogs participating in the study had at least a Class 0 certification, allowing for further training in rescue work. The dogs are certified by the School Aspirants for the State Fire Service, Specialist Rescue Team Training Department. The dogs trained at least twice a week. All dogs were clinically healthy, free of chronic diseases, and not on any ongoing medication. The dogs were up to date on vaccinations and received standard preventive care. The dogs were fed in accordance with nutritional standards for search and rescue dogs in Poland. The handlers declared good health, no chronic diseases, and no ailments that could affect their ability to train or participate in rescue operations.

Belgian Shepherds (Malinois) comprised the largest population of dogs studied, with 15 (25.0%), followed by Labrador Retrievers with 11 (18.3%). Dogs of other breeds were next in number, totalling 9 (15.0%). German Shepherds accounted for 8 (13.3%), while Border Collies and mixed-breed dogs each had 6 (10.0%). Golden Retrievers were the smallest group, with 5 (8.3%). The breed composition reflects the requirements of rescue work, which favours dogs with high endurance, high motivation, and the ability to cooperate with their handler. Regarding gender, 33 females (55.0%) and 27 males (45.0%) participated in the study. Of the population, 31 dogs (51.7%) were intact, while 29 (48.3%) had been castrated or spayed. The age distribution of the dogs was uneven—the largest group consisted of dogs aged 2–3 years (*n* = 26), followed by dogs aged 4–6 years (*n* = 21), while the smallest group consisted of dogs aged 7 years and older (*n* = 13). Therefore, the largest group consisted of young adult dogs (approximately 2–3 years old), which corresponds to the period when the animal reaches physical and behavioural maturity appropriate for intensive work.

The study material consisted of hair from rescue dogs and their handlers. Hair was collected from the dogs’ interscapular region and from the handlers’ occipital region. The collection was non-invasive and involved cutting a section of hair directly from the skin using clean scissors. The hair was placed in plastic zip-lock bags, protected from moisture, and stored at room temperature, away from sunlight, until analysis. A section approximately 1 cm long from the hair root was used for analysis, representing an average of 1 month of hormone exposure. Cortisol (HCL) and testosterone (HTL) levels were determined in the collected biological material. The cortisol/testosterone ratio (C/T) was also calculated.

Extraction methodology was taken from Koren et al. [20] and Accorsi et al. [21]. Hair was first minced into 1–2 mm length fragments and 20 mg of trimmed hair were put in a glass vial. Three and a half mL methanol (Sigma-Aldrich, Poznań, Poland) were added, and vials were incubated at 50 °C with gentle shaking for 24 h. After incubation, the supernatant was filtrated to separate the liquid phase and put into disposable glass culture tubes. Next, this supernatant was evaporated to dryness under an air-stream suction hood at 37 °C. The dry residue was reconstituted in 1 mL of phosphate-buffered saline (PBS, 0.05 M, pH 7.5) and thoroughly mixed by vortexing for 1 min followed by an additional 30 s. Cortisol and testosterone concentrations were quantified using commercial ELISA kits (Bioassay Technology Laboratory, Shanghai, China and Reed Biotech, Wuhan, China, respectively), according to the manufacturers’ instructions. Hormone concentrations were expressed as pg/mg of hair.

Statistical analyses were performed using Statistica 13.3 (TIBCO Software Inc., Palo Alto, CA, USA). Data distribution was assessed using the Shapiro–Wilk test. As the assumptions of normality and homogeneity of variance were not met, nonparametric tests were applied.

Spearman’s rank correlation was used to assess the relationships between quantitative variables (cortisol and testosterone levels in dogs and their handlers, and the C/T ratio). Differences between the two groups (e.g., dog gender, neutering status, presence of other dogs in the home, kennel use, and handler gender) were analysed using the Mann–Whitney U test with a continuity correction. For categorical variables encompassing more than two groups (e.g., breed, age of dog), the Kruskal–Wallis ANOVA test was used. For significant differences, post hoc comparisons were performed using the Dunn-Bonferroni method. Results are presented as means ± standard deviation (SD). All analyses were performed at a significance level of *p* < 0.05.

## 3. Results

The mean HCL in handlers was 10.874 pg/mg (SD = 0.399), the mean HTL was 2.925 pg/mg (SD = 0.569), and the C/T ratio was 3.875 (SD = 0.852). In dogs, the mean HCL was 10.974 pg/mg (SD = 1.191), the mean HTL was 3.008 pg/mg (SD = 0.516), and the C/T ratio was 3.795 pg/mg (SD = 1.054). The values of the measured parameters obtained in the study group by gender are summarised in Table 1.

**Figure 1:**
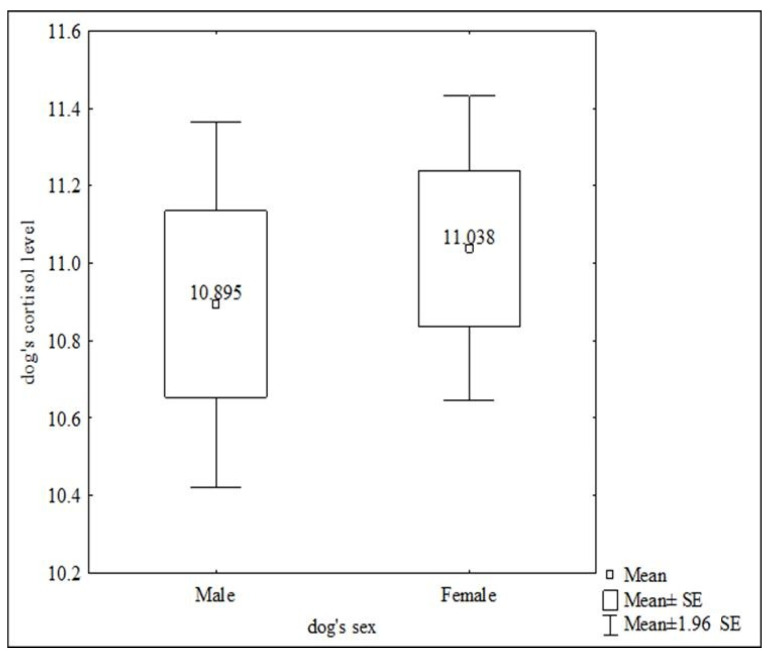
Dog’s hair cortisol level depending on the dog’s sex.

Female dogs had significantly higher hair cortisol levels than male dogs (Z = −2.0060; *p* = 0.0449) (Figure 1). No such difference was observed in testosterone levels (Z = 0.5498; *p* = 0.5825). No significant differences were found between the sexes of the handlers in either HCL levels (Z = −0.4485; *p* = 0.6538) or HTL levels (Z = 1.0795; *p* = 0.2804).

**Figure 2:**
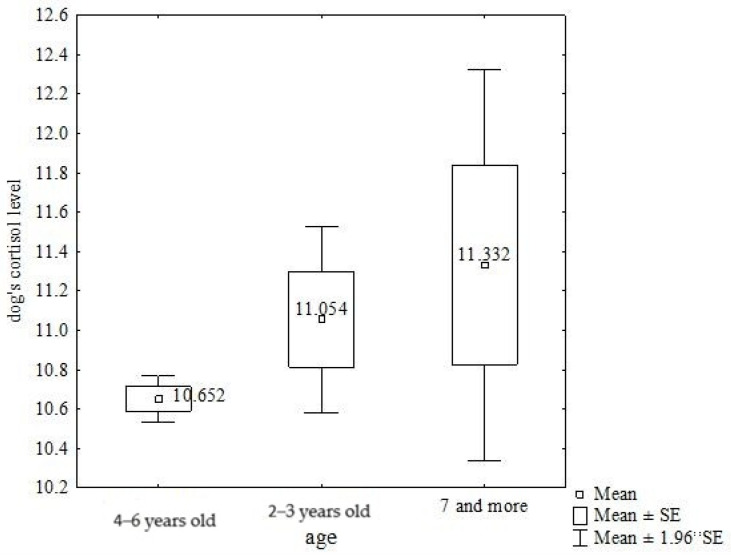
Dog’s hair cortisol level depending on the dog’s age.

There was a trend toward differences in cortisol levels in dogs based on age (H = 5.495626; *p* = 0.064). The older the dog, the higher the hair cortisol level (Figure 2). No such relationship was observed in handlers. There were no significant differences in cortisol or testosterone levels in the hair of dogs based on castration status (HCL Z = −0.3994; *p* = 0.6896; HTL Z = 0.5177; *p* = 0.6046).

The division of dogs by breed did not reflect differences in cortisol levels (H = 4.5065; *p* = 0.6085) or testosterone levels (H = 4.3319; *p* = 0.6319). Interestingly, dog breed had a significant effect on cortisol levels, but in the handler’s hair, not the dog’s (H = 15.49651; *p* = 0.0167). The lowest mean cortisol levels were recorded in handlers of German Shepherd dogs (10.493 pg/mg), while the highest were observed in handlers of mixed-breed dogs (pg/mg). Neither the handler’s gender nor the dog’s sex, breed, or age affected the C/T ratio. A significant negative correlation was found between HCL and HTL levels in both dogs (Z = −0.317; *p* = 0.014) and their handlers (Z = −0.284; *p* = 0.028). A significant negative correlation was observed between cortisol levels in dogs and their handlers (Z = −0.293; *p* = 0.023).

## 4. Discussion

The mean hair cortisol level of search-and-rescue dog handlers in our study was 10,874 pg/mg. No significant differences were found based on handler gender. The mean cortisol level in paramedics in the study by Johnsen et al. [22] was 19.2 pg/mg and did not differ between men and women. Similarly, among the paramedics examined in the study [23], there was no difference in mean cortisol levels between men (13.0 pg/mg) and women (14.8 pg/mg). Abell et al. [13] also found no differences in hair cortisol levels between men and women. For comparison, in the study by Faresjö et al. [24], men had significantly higher HCL levels (median 32.6 pg/mg) than women (median 25.1 pg/mg). We did not observe an effect of handler age on hair cortisol levels. The study by Faresjö et al. [24] also found no significant difference in HCL with age. However, the survey by Pulopulos et al. [25] showed that in older individuals, higher HCC levels are associated with better cognitive performance (including working memory, learning, and short- and long-term verbal memory). Hair cortisol concentration also directly correlates with subjectively perceived stress [26,27].

Cortisol is one of the most frequently measured hormones in animal hair. The mean cortisol level in the hair of rescue dogs in our sample was 10,974 pg/mg. For comparison, the study by van Houter et al. [28] found that service dogs had a mean concentration of 9.69 pg/mg, while companion dogs had a mean concentration of 8.65 pg/mg, with no statistically significant difference between groups. In the study by Mariti et al. [29], healthy pet dogs had a mean HCL of 6.41 pg/mg. These results suggest that the nature of their work—requiring constant readiness and high responsiveness—significantly influences long-term cortisol levels in SAR dogs. At the same time, this does not indicate chronic stress exceeding levels observed in other working dog groups, suggesting adequate preparation of these dogs for SAR tasks, practical training, and proper selection of individuals.

We observed a statistically significant difference in HCL between females and males. In the study by Schell et al. [30], male coyotes had significantly higher cortisol concentrations than females.

The Spearman correlation between HCL and dog age in the study by Mariti et al. [29] (S = 287,258; *p* = 0.0442) was statistically significant. Mesarcova et al. [27] also observed an age-related increase in HCL in dogs. In our study, we observed only a trend toward higher cortisol levels in older dogs compared to younger ones.

Comparing the dogs’ environmental conditions, the study by van Houter et al. [28] found no significant differences in hair cortisol levels based on coat type (black/brown or blond; *p* = 0.63), the presence of other dogs (*p* = 0.72), or the presence of other pets in the household (*p* = 0.92). Our results were consistent with these observations—we observed no significant differences in HCL or HTL depending on whether more than one dog lived in the household (Z = 0.6398, *p* = 0.5223; Z = 0.2707, *p* = 0.7866). Contrary results were obtained by Packer et al. [31], who found that dogs living with three or more other dogs had significantly higher HCL levels than dogs living with only one other dog (*p* = 0.018) or without other dogs (*p* = 0.042).

Similar to cortisol, testosterone also accumulates in hair during hair growth, making this valuable matrix for assessing chronic, rather than merely short-term, hormonal changes [16,32]. The mean HTL of rescue dog handlers in our study was 2.925 pg/mg (SD = 0.569). In women, it was 2.866 pg/mg on average, and in men, 3.019 pg/mg. In the study by Kintz et al. [33], the mean testosterone level in the studied group of men was 3.8 pg/mg. In the study by Deshmukh et al. [34], the mean testosterone level in men’s hair was 2.67 pg/mg, and in women, 1.62 pg/mg. They also observed that testosterone levels in men were significantly higher than in women (*p* = 0.020). In the study by Shen et al. [33], physiological testosterone concentrations ranged from 0.8 to 24.2 pg/mg, 0.1 to 16.8 pg/mg, and 0.2 to 11.5 pg/mg in men, women, and children, respectively. The range of testosterone levels in the study groups was therefore quite significant.

Yang et al. [35] found that testosterone levels in males were strongly correlated with serum testosterone levels. This relationship was not found in the study by Calamari et al. [36], among others.

The average testosterone level in the hair of SAR dogs in our sample was 3.008 pg/mg. For comparison, the study by Fusi et al. [37] found an average HTL of 2.87 pg/mg in adult Doberman Pinschers, which is similar to the values obtained in our sample. Due to the limited number of studies on testosterone in dog hair, direct comparisons of results are difficult.

We did not observe significant differences in HTL between sexes or reproductive statuses in our canine sample. While Calamari et al. [36] demonstrated significant sex-based dimorphism in canine hair testosterone concentrations, our sample did not exhibit such variance. This lack of differentiation in our dataset may be attributed to sample size constraints, the uniform training regimes of professional lines, or local tissue metabolism within the hair follicle. Therefore, the uniform testosterone levels observed in our search and rescue (SAR) study population may reflect population-specific characteristics associated with long-term occupational demands rather than general population endocrine patterns [7,8]. Although testosterone is primarily regulated by gonadal activity, hair testosterone likely reflects integrated long-term endocrine dynamics influenced by multiple physiological systems [16,32]. The absence of expected sex- or reproductive-status-related differences suggests that chronic environmental and occupational factors may modulate endocrine variability in working dogs [7,8]. However, the exact mechanisms remain unclear. These discrepancies further highlight that hair hormone concentrations may not directly mirror circulating hormone levels across all contexts and populations. A significant finding in the literature is the lack of a simple relationship between hair testosterone concentrations and circulating testosterone levels [36].

An essential point of reference for interpreting the results of this study is the Dual-Hormone Hypothesis (DH), which posits that the effects of testosterone on status, risk-taking, and proactive behaviours are modulated by cortisol levels [38]. In rock hyraxes, individuals with high testosterone and low cortisol levels were more likely to exhibit leadership and proactive behaviours [39]. Conversely, in macaques used for entertainment purposes, chronically elevated cortisol and decreased testosterone were indicators of HPA/HPG axis imbalance and poor well-being [32]. This pattern, high cortisol combined with low testosterone, is considered characteristic of organisms under chronic stress. In the context of search-and-rescue (SAR) teams, the relationship between testosterone and cortisol reflects homeostatic axis crosstalk rather than individual personality traits. Because our dataset demonstrated that the cortisol-to-testosterone (C/T) ratio remained statistically uniform across all canine demographics, dog breeds, and age groups, this metric should not be interpreted as a tool to differentiate individual “proactive” or “reactive” coping styles or behavioral selection traits within this population. Instead, the uniform negative correlation observed between canine cortisol and testosterone levels may reflect central homeostatic feedback processes. Testosterone may exert negative feedback effects at the hypothalamic level, contributing to modulation of CRH-related signaling and subsequently influencing downstream adrenal cortisol output. Therefore, the C/T ratio in this study appears to reflect the overall balance of these interacting endocrine systems under high-amplitude occupational demands. It should be explicitly acknowledged that the application of the C/T ratio within a canine hair matrix remains a preliminary, unvalidated exploratory metric rather than an established diagnostic tool for animal welfare or behavioral performance.

It is also important to emphasize that testosterone in dogs is not necessarily directly linked to aggression or dominance, as has often been suggested in older literature. Studies involving dogs indicate that testosterone influences social motivation, willingness to explore, arousal, and situational assessment rather than aggression itself [40]. Therefore, its potential role in rescue dog work may be more related to maintaining work readiness and initiative than to conflict behavior. From a working-dog perspective, testosterone may be relevant because its biological role extends beyond reproduction and aggression. According to the Dual-Hormone Hypothesis, individuals characterised by relatively higher testosterone concentrations in combination with lower cortisol levels may be more likely to display proactive behavioural tendencies, leadership-related behaviours, and increased willingness to engage with environmental challenges [10,39]. Whether such endocrine profiles are associated with operational performance in SAR dogs remains to be determined. Recent studies have further highlighted that successful SAR performance depends not only on olfactory capabilities but also on behavioural traits such as trainability, resilience, motivation, and adaptability to challenging operational environments [41,42]. Although direct evidence linking hair testosterone concentrations to SAR performance is currently lacking, long-term testosterone profiles may represent a promising physiological indicator of behavioural traits relevant to operational readiness and working success, warranting further investigation.

Between dogs and their handlers, stress might be emotionally shared, and their bond can modulate the hormonal response to stress. A shared short-term testosterone response was evident in agility teams [43], and short-term stress was previously described in SAR teams [44]. Although stress synchronisation between animals and their handlers is apparent, it can be modulated by attachment, training style, or trust between both parties. In the handlers, their stress reaction can be moderated by individual differences [10], such as experience, gender, temperament, and risk assessment during action. Research on hormonal interactions in human–human and human–animal pairs is increasingly of interest to scientists. For example, a positive correlation between mother and child HCL was found [45,46]. In our previous studies, we looked for such a correlation between animals and their owners. We found no significant correlation between cortisol levels in the hair of owners and dogs, or between owners and cats, or between dogs and cats living together [47]. Sundman et al. [48] found a significant correlation between cortisol levels in the hair of dogs and their owners. As HCL levels increased in humans, they observed a similar increase in their dogs. However, in subsequent studies, these authors did not confirm this relationship [49]. In our study, we observed a significant but negative correlation between cortisol levels in dogs and their owners. However, as emphasized by Mariti et al. [29], raw cortisol concentrations should not be interpreted in isolation, as hair hormone levels are influenced by multiple factors related to both handler characteristics and environmental conditions.

We did not find that the sex of the handler, nor the sex, breed, or age of the dog, influenced the C/T ratio. In comparison, in the study by Chan et al. [50], the hair C/T ratio significantly correlated with age, BMI, and waist circumference. Pereg et al. [51] analysed the usefulness of the C/T ratio as a biological marker of systolic heart failure, and Chan et al. [50] did so in the context of obesity. In their studies, the C/T ratio correlated better with the studied factors than hair cortisol or testosterone levels alone. Interpreting hair hormone levels must be done with caution, mainly since their levels can be influenced by confounding factors such as hair colour, hair growth rate, and local hormone production in hair follicles [52,53,54,55]. Although local synthesis of hormones, including cortisol, has been confirmed in guinea pigs and sheep [52,54], most available data indicate that circulating hormones remain the dominant source. Nevertheless, these factors must be considered when interpreting testosterone concentrations in dogs with different coat types and colours. In a study by Preinbergs et al. [56], hair treatments in humans, such as dyeing or bleaching, were associated with increased testosterone concentrations (*p* = 0.051). In contrast, natural hair colour did not affect testosterone levels (*p* = 0.133). According to some authors, the body part from which the hair was collected may also influence hormone levels [57,58]. For comparison, in the study by Schell et al. [30], no differences in cortisol or testosterone concentrations were observed across hair collection sites (e.g., back, belly, tail, shoulders).

Several limitations of this field study should be acknowledged. First, although factors such as operational history, deployment frequency, both canine and handler experience and household environment may influence long-term endocrine profiles, our statistical approach focused on core handler–dog demographic variables to preserve analytical robustness within this relatively small working population. Second, intact and gonadectomized dogs were analysed together due to sample size constraints typical of active search-and-rescue (SAR) teams. While this approach may mask subtle subgroup differences, it allows for a more stable evaluation of overall endocrine patterns associated with SAR work. Future studies with larger datasets should consider stratified analyses to further explore the effects of reproductive status and other physiological modifiers.

## 5. Conclusions

This study provides new information on the long-term hormonal profiles of search and rescue (SAR) dogs and their handlers, as assessed by hair cortisol and testosterone concentrations. These results confirm that both species exhibit significant interindividual variability in hormonal activity, but common patterns emerge within the working dyad.

The dogs exhibited cortisol levels similar to those of other working dog populations, with higher values in females and a tendency to increase with age. Testosterone concentrations did not differ between sexes, suggesting that hair testosterone may reflect long-term HPG axis regulation rather than short-term circulating hormone concentrations.

The dog handlers exhibited hair hormone concentrations comparable to those of other high-risk professions, with no gender differences. Significantly, cortisol levels in the handlers varied by dog breed. In both species, cortisol and testosterone were negatively correlated, consistent with the dual-hormone hypothesis. The negative correlation between cortisol in dogs and handlers suggests that long-term physiological dynamics in SAR syndromes do not reflect the positive stress synchrony reported in some acute response studies. In summary, hair hormone analysis is a valuable tool for assessing long-term physiological status in SAR syndromes. These findings highlight the need to integrate indicators of the HPA and HPG axes when assessing the well-being, training, and performance of working dog–handler partnerships.

## Figures and Tables

**Table 1:** Hair cortisol and testosterone levels in dogs and their handlers.

Parameter	Group	Mean	Min	Max	SD
Hair cortisol level (pg/mg)	Male dogs	10.895	9.906	16.891	1.251
	Female dogs	11.038	10.021	17.270	1.154
	Male handlers	10.989	9.906	16.891	1.332
	Female handlers	10.964	10.113	17.270	1.114
Hair testosterone level (pg/mg)	Male dogs	3.067	2.186	3.954	0.507
	Female dogs	2.960	1.850	3.714	0.526
	Male handlers	3.019	1.702	3.741	0.613
	Female handlers	2.866	2.080	4.135	0.540

## Data Availability

The data presented in this study are available on request from the corresponding authors.

## Abbreviations

The following abbreviations are used in this manuscript:

SAR	Search and Rescue
HCL	Hair Cortisol Level
HTL	Hair Testosterone Level
